# Supernumerary Renal Arteries and Their Embryological and Clinical Correlation: A Cadaveric Study from North India

**DOI:** 10.5402/2013/405712

**Published:** 2013-02-20

**Authors:** Virendra Budhiraja, Rakhi Rastogi, Vaibhav Anjankar, C. S. Ramesh Babu, Prabhat Goel

**Affiliations:** ^1^Department of Anatomy, L. N. Medical College and Research Center, Bhopal, India; ^2^Department of Anatomy, Muzaffarnagar Medical College, Muzaffarnagar, India; ^3^Department of Anatomy, Subharti Medical College, Meerut, India

## Abstract

*Background*. Classically, each kidney is supplied by a single renal artery originating from abdominal aorta. The present study aimed at its variations and their embryological and clinical correlation. *Material and Methods*. The formalin-fixed thirty-seven cadavers from north India constituted the material for the study. During routine abdominal dissection conducted for medical undergraduates at the department of anatomy, the kidneys along with their arteries were explored and the morphological variations of renal arteries were noted. *Results*. We observed supernumerary renal arteries in 23/37 (62.2%) cases (48.6% of aortic origin and 13.5% of renal origin) on the right side and 21/37 (56.8%) cases (45.9% of aortic origin and 10.8% of renal origin) on the left side. Supernumerary renal arteries entered the kidney through hilum, superior pole, and inferior pole. *Conclusion*. Awareness of variations of renal artery is necessary for surgical management during renal transplantation, repair of abdominal aorta aneurysm, and urological procedures and for angiographic interventions.

## 1. Introduction

Classically, the description of a single renal artery arising from abdominal aorta that supplies the respective kidney [1, 2] occurs in less than 25% of cases [3, 4]. Common variations of renal artery are its variable number and unusual branching pattern [5–9]. Variations in renal arteries have been called aberrant, supplementary, and accessory, among other terms. We used the term supernumerary and analyze it in accordance with Merklin classification [10]. We believe that prior knowledge of these possible variations of renal arteries may help the surgeon in planning renal transplantation, repair of abdominal aorta aneurysm, urological procedures, and also for angiographic interventions [11–13].

## 2. Materials and Methods

The formalin-fixed thirty-seven cadavers constituted the material for the study. During routine abdominal dissection conducted for medical undergraduates at the department of anatomy, the kidneys along with their arteries were explored and the morphological variations of renal arteries were noted. During the course of dissection various abdominal viscera were removed and preserved as specimens for teaching purposes. We studied the origin of supernumerary renal arteries in accordance to the nomenclature of Merklin and Michels [10]:

1. supernumerary renal arteries originating from the aorta;
2. supernumerary renal arteries originating from main renal arteries;
3. supernumerary renal arteries that can come from other sources.

## 3. Results

**Figure 1:**
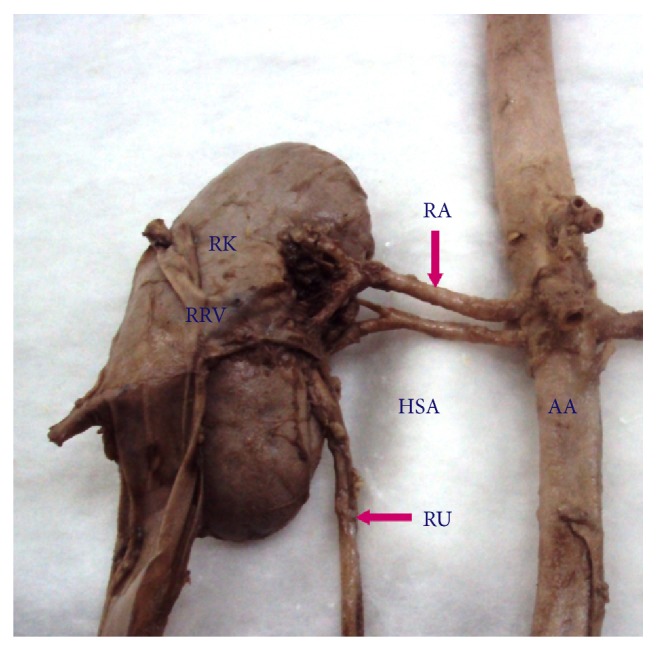
Right kidney showing hilar supernumerary renal artery originating from abdominal aorta. AA: abdominal aorta, RA: renal artery, HSA: hilar supernumerary renal artery, RK: right kidney, RU: right ureter, RRV: reflected renal vein.

**Figure 2:**
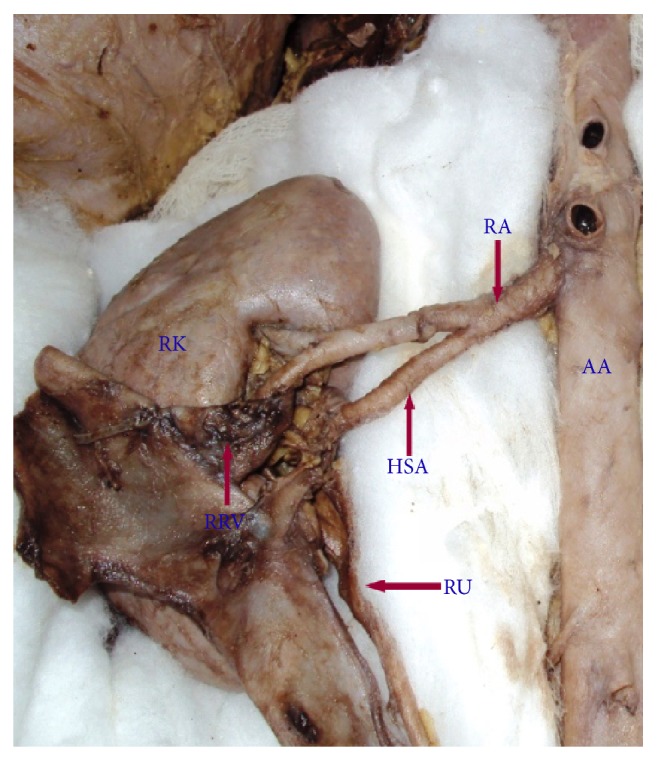
Right kidney showing hilar supernumerary renal artery originating from renal artery. AA: abdominal aorta, RA: renal artery, HSA: hilar supernumerary renal artery, RK: right kidney, RU: right ureter, RRV: reflected renal vein.

**Figure 3:**
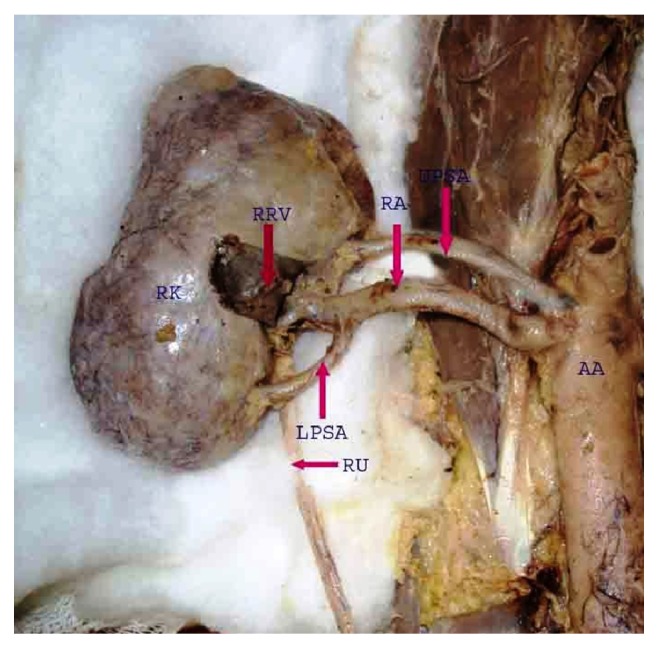
Right kidney showing upper polar supernumerary renal artery originating from abdominal aorta and lower polar supernumerary renal artery originating from renal artery. AA: abdominal aorta, RA: renal artery, UPSA: upper polar supernumerary renal artery, LPSA: lower polar supernumerary renal artery, RK: right kidney, RU: right ureter, RRV: reflected renal vein.

**Figure 4:**
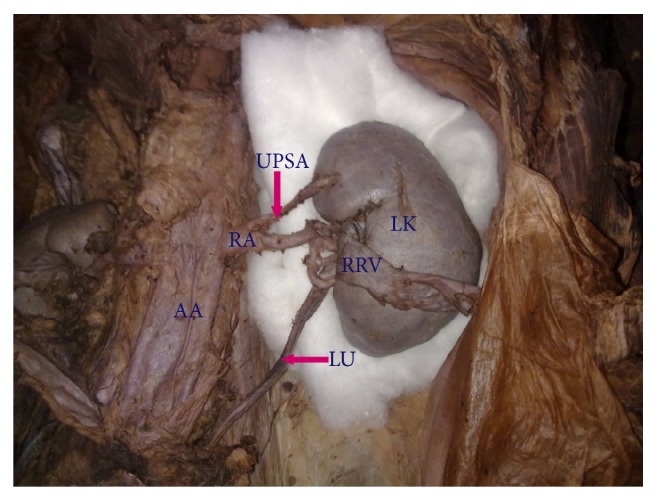
Left kidney showing upper polar supernumerary renal artery originating from renal artery. AA: abdominal aorta, RA: renal artery, UPSA: upper polar supernumerary renal artery, LK: left kidney, LU: left ureter, RRV: reflected renal vein.

**Figure 5:**
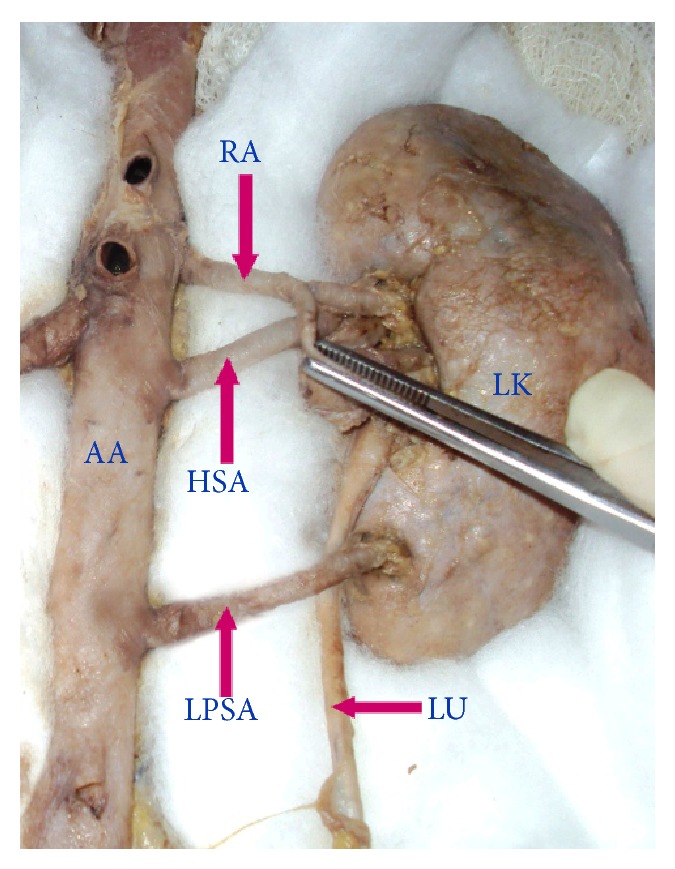
Left kidney showing hilar supernumerary renal artery and lower polar supernumerary renal artery originating from abdominal aorta. AA: abdominal aorta, RA: renal artery, HSA: hilar supernumerary renal artery, LPSA: lower polar supernumerary renal artery, LK: left kidney, LU: left ureter.

Supernumerary renal arteries were present in 23/37 (62.2%) cases (48.6% of aortic origin and 13.5% of renal origin) on the right side and 21/37 (56.8%) cases (45.9% of aortic origin and 10.8% of renal origin) on the left side. The supernumerary renal arteries entered the kidney through hilum as hilar supernumerary renal artery (Figures 1, 2, and 5), through upper pole as upper polar supernumerary renal artery (Figures 3 and 4), and through lower pole as lower polar supernumerary renal artery (Figures 3 and 5). The finding with respect to origin, side, and mode of penetration to kidney is represented in Table 1. We did not find any case where supernumerary renal artery was originating from other source like common iliac, testicular, ovarian, suprarenal, and so forth.

## 4. Discussion

The various types of (accessory, additional, supplementary, and aberrant) renal arteries, their positions, method of entry to the kidney, and segmentation were studied extensively by a number of authors [14, 15] but the generally accepted and precise terminology for these arteries has not been unified by the majority of authors [16]. As these arteries occupy a certain vascular area within the kidney and there is no anastomosis, either with the branches of the main or with branches of segmental renal arteries, we preferred the terminology supernumerary for these arteries and classified them in accordance with Merklin and Michels [10]. Merklin and Michels [10] classified these supernumerary renal arteries depending upon origin as supernumerary renal arteries originating from aorta, supernumerary renal arteries originating from the main renal artery, and supernumerary renal arteries originating from other arterial sources, but in their study none of the hilar supernumerary renal artery took origin from renal artery. Talovic et al. [17] reported that in 30.76% cases supernumerary renal arteries originated from aorta and in 12.82% originated from renal arteries. In the present study the supernumerary arteries originated from abdominal aorta in 47.3% cases and from the main renal artery in 12.2% cases. We also observed hilar supernumerary renal arteries originating from renal artery in 8.1% cases.

Embryological explanation of these variations has been presented and discussed by Felix [18]. In an 18 mm fetus, the developing mesonephros, metanephros, suprarenal glands, and gonads are supplied by nine pairs of lateral mesonephric arteries arising from the dorsal aorta. Felix divided these arteries into three groups as follows: the 1st and 2nd arteries as the cranial, the 3rd to 5th arteries as the middle, and the 6th to 9th arteries as the caudal group. The middle group gives rise to the renal arteries. Persistence of more than one artery of the middle group results in multiple renal arteries [18]. Thus, the multiple renal arteries in our study are a result of persisting lateral mesonephric arteries from the middle group.

Clinically, the supernumerary renal arteries are very important. Upper polar and lower polar supernumerary renal arteries originating from renal arteries, directed towards superior or inferior pole, have vertical trajectory in comparison to supernumerary renal arteries taking origin from aorta. Vertical trajectory of these arteries can lead to polar infarction [19] and they can also be injured during mobilization and other surgical procedures [9]. Lower polar supernumerary renal arteries of aortic or renal origin can be a cause of ureteropelvic junction obstruction [20].

The anatomical knowledge of supernumerary renal arteries is essential before performing any transplantation surgeries where microvascular techniques are employed to reconstruct the renal arteries [21]. One has to keep in mind that transplanting a kidney with accessory renal arteries has several theoretical disadvantages: acute tubular necrosis and rejection episodes and decreased graft function [9].

We believe that awareness of variations is necessary for surgical management during renal transplantation, repair of abdominal aorta aneurysm, and urological procedures and for angiographic interventions.

## Figures and Tables

**Table 1:** Number, percentage, and types of supernumerary renal arteries.

Number of renal artery	Right kidney (%)	Left kidney (%)	Total (%)
One artery	14/37 (37.8%)	16/37 (43.2%)	30/74 (40.5%)
Supernumerary renal artery	23/37 (62.2%)	21/37 (56.8%)	44/74 (59.5%)
(A) Aorta origin	**18/37** (48.6%)	**17/37** (45.9%)	**35/74** (47.3%)
HSA	09/37 (24.3%)	07/37 (18.9%)	16/74 (21.6%)
UPSA	06/37 (16.2%)	05/37 (13.5%)	11/74 (14.9%)
LPSA	03/37 (8.1%)	05/37 (13.5%)	08/74 (10.8%)
(B) Renal origi	**05/37** (13.5%)	**04/37** (10.8%)	**09/74** (12.2%)
HSA	03/37 (8.1%)	03/37 (8.1%)	06/74 (8.1%)
UPSA	01/37 (2.7%)	01/37 (2.7%)	02/74 (2.7%)
LPSA	01/37 (2.7%)	0/37 (0%)	01/74 (1.4%)

HSA: hilar supernumerary renal artery, UPSA: upper polar supernumerary renal artery, LPSA: lower polar supernumerary renal artery.
